# Distinct hemodynamic implications of junctional rhythm versus sinus rhythm on cardiac reverse remodeling after the Maze procedure

**DOI:** 10.1186/s13019-026-04550-7

**Published:** 2026-07-22

**Authors:** Hee Jeong Lee, YoHan Bae, Jung Uk Woo, Sungsil Yoon, Woo Sung Jang, Yun Seok Kim, Jonghoon Yoo, Kyungsub Song

**Affiliations:** 1 Department of Cardiology, Keimyung University Dongsan Hospital, Keimyung University College of Medicine, Daegu, Republic of Korea; 2 Department of Thoracic and Cardiovascular Surgery, Keimyung University Dongsan Hospital, Keimyung University College of Medicine, Daegu, 42601 Republic of Korea; 3 Medical Course, School of Medicine, Keimyung University College of Medicine, Daegu, Republic of Korea; 4 Department of Emergency Medicine, Keimyung University Dongsan Hospital, Keimyung University College of Medicine, Daegu, Republic of Korea

**Keywords:** Atrial fibrillation, Cox-Maze procedure, Atrial function, Junctional rhythm, Pacemaker, Cardiac remodeling

## Abstract

**Background:**

Surgical success after the Maze procedure is conventionally defined as “freedom from atrial fibrillation (AF),” often grouping junctional rhythm (JR) with normal sinus rhythm (NSR). However, the specific hemodynamic impact of JR remains poorly understood. This study investigated whether JR truly represents a successful outcome compared to NSR and AF.

**Methods:**

We retrospectively analyzed patients undergoing the Maze procedure (2012–2024). After designating the first postoperative month as a blanking period for rhythm instability, we included only patients maintaining a consistent rhythm without crossover from 1 to 12 months postoperatively: AF (*n* = 71), NSR (*n* = 213), and JR (*n* = 35). Outcomes included cardiac reverse remodeling—assessed by left atrial contractile strain (LASct) and chamber dimensions—and permanent pacemaker (PPM) implantation.

**Results:**

In the IPTW-adjusted cohort, the NSR group achieved significant cardiac reverse remodeling, characterized by improved LVEF (58.5%), reduced LA volume index (75.1 mL/m²), and notable recovery of LASct. In stark contrast, the JR group failed to show any reverse remodeling or mechanical recovery, exhibiting hemodynamic stagnation indistinguishable from the AF group. Consequently, no significant difference in cardiac function was observed between the JR and AF groups. Furthermore, the JR group faced a drastically elevated risk of PPM implantation compared to both the NSR and AF groups (*P* < 0.001).

**Conclusions:**

JR is not a benign alternative to sinus rhythm; it mimics AF in preventing cardiac reverse remodeling and carries a high risk of pacemaker dependency. Therefore, JR should be clinically differentiated from NSR and recognized as a suboptimal hemodynamic outcome similar to AF.

**Supplementary Information:**

The online version contains supplementary material available at https://doi.org/10.1186/s13019-026-04550-7.

## Background

The Cox-Maze procedure is established as the gold standard for treating drug-refractory atrial fibrillation (AF) [1, 2]. Conventionally, surgical success is defined as “freedom from AF,” a binary classification that often categorizes postoperative junctional rhythm (JR) as a successful outcome alongside normal sinus rhythm (NSR) [3, 4]. However, physiologically, these rhythms represent fundamentally different hemodynamic states. While NSR preserves atrioventricular synchrony and the “atrial kick,” JR—driven by a subsidiary pacemaker—implies the loss of coordinated atrial contraction. This can lead to adverse hemodynamics, such as Cannon A waves and reduced cardiac output, as well as a significantly elevated risk of requiring permanent pacemaker (PPM) implantation [5–7].

Recently, left atrial strain (LAS) using speckle-tracking echocardiography has emerged as a superior marker for detecting functional reverse remodeling compared to traditional volumetric indices [8, 9]. Emerging evidence suggests that mechanical recovery of the atrium is a more critical determinant of prognosis than simple electrical maintenance of non-AF rhythm [10]. Despite this, a significant gap remains in current literature: most studies aggregate JR and NSR into a single “sinus conversion” or “success” group based on current guidelines, obscuring the specific hemodynamic and prognostic implications of JR [3, 11, 12].

Therefore, this study aims to scrutinize the impact of postoperative rhythm—specifically differentiating JR from NSR—on cardiac reverse remodeling and clinical outcomes. We focused on LA contractile strain as an indicator of mechanical recovery and analyzed its association with long-term adverse events, including permanent pacemaker implantation.

## Methods

### Ethical statement

The Institutional Review Board of the Dongsan Medical Center approved the study (IRB File No. 2025-11-062, November 25, 2025). Written informed consent was waived by Medical Ethics Committee of the Dongsan Medical Center, Keimyung University School of Medicine because this was a retrospective study. All methods were carried out in accordance with relevant guidelines and regulations.

### Study population and design

This single-center, retrospective study included patients who underwent the Maze procedure at our institution between July 2012 and March 2025. To evaluate the chronic impact of a sustained rhythm status on cardiac structure and function, we included only patients who maintained a consistent cardiac rhythm without crossover from 1 month to 12 months postoperatively. The first postoperative month was designated as a “post-operative rhythm unstable state” and served as a blanking period to account for transient rhythm fluctuations commonly observed in the early recovery phase.

The exclusion criteria were as follows: (1) age less than 18 years; (2) mortality within one year postoperatively; (3) incomplete or missing echocardiographic data; (4) rhythm crossover among AF, JR, and NSR between 1 and 12 months postoperatively; and (5) loss to follow-up.

Based on serial 12-lead electrocardiograms (ECGs) and 24-hour Holter monitoring results obtained from 1 to 12 months postoperatively, the final study population was categorized into three groups: Atrial Fibrillation (AF, *n* = 71), Normal Sinus Rhythm (NSR, *n* = 213), and Junctional Rhythm (JR, *n* = 35).

### Operative techniques of Maze surgery

A modified Cox-Maze procedure was performed using cryoablation under antegrade or retrograde cardioplegia, following established principles [13]. Patient selection relied on predictors of rhythm restoration, including LA characteristics and AF duration. The LA lesion set comprised pulmonary vein isolation and a mitral isthmus line. The RA lesion set included an intercaval line, a ‘T’ lesion to the tricuspid annulus, and a line involving the right atrial appendage. Left atrial appendage obliteration was mandatory for patients with a history of stroke or thrombus, and otherwise performed at the surgeon’s discretion.

### Echocardiographic assessment

Comprehensive transthoracic echocardiography (TTE) was performed preoperatively and at one year postoperatively using commercially available ultrasound systems (Vivid E9/E95, GE Healthcare, Horten, Norway). All measurements followed the American Society of Echocardiography (ASE) guidelines [14]. Standard parameters included left ventricular ejection fraction (LVEF), left atrial volume index (LAVI), left atrial diameter (LAD), and left ventricular end-diastolic dimension (LVEDD).

Speckle-tracking echocardiography (STE) was used to assess left atrial strain from the apical four-chamber view. LA reservoir strain (LASr), conduit strain (LAScd), and contractile strain (LASct) were measured using vendor-specific software (EchoPAC, GE Healthcare). LASct, reflecting active atrial contraction (“booster pump” function), was the primary functional endpoint. For LASct, more negative values indicate stronger contractile function.

### Rhythm classification

Cardiac rhythm was evaluated using serial 12-lead ECGs and 24-hour Holter monitoring at each follow-up visit (1, 3, 6, and 12 months postoperatively). The first postoperative month was designated as a blanking period to account for the post-operative rhythm unstable state. NSR was strictly defined as a regular rhythm originating from the sinus node, with a normal P-wave axis and a resting ventricular rate of 60–100 beats per minute. Patients with sinus bradycardia, sinus tachycardia, or sinus arrhythmia were not classified as NSR, and rhythms other than AF, JR, and NSR were excluded from the study. AF was classified according to 2020 European Society of Cardiology (ESC) guidelines as paroxysmal or persistent [15]. JR was diagnosed when a narrow QRS complex rhythm was present without preceding P waves, with a ventricular rate typically between 40 and 60 beats per minute [16]. Only patients who maintained a single, consistent rhythm classification from 1 to 12 months postoperatively were included in the analysis.

### Statistical analysis

To address the inherent selection bias in this observational study, we employed inverse probability of treatment weighting (IPTW) using a multinomial logistic regression model. Covariates for the propensity score model included AF type (persistent vs. paroxysmal), preoperative LVEF, preoperative LAVI, and preoperative LVEDD. Stabilized weights were calculated as 1/P(G|X), where G is the assigned group and X is the covariate vector. Weights were normalized within each group, and covariate balance after weighting was assessed using standardized mean differences (SMD), with values < 0.2 considered well-balanced.

Continuous variables were expressed as mean ± standard deviation and compared using weighted t-tests. Categorical variables were expressed as frequencies and percentages and compared using chi-squared tests or Fisher’s exact test. All pairwise comparisons among the three groups (AF vs. NSR, NSR vs. JR, AF vs. JR) were performed with corresponding P-values reported. Freedom from PPM implantation and MACE was analyzed using Kaplan–Meier survival curves with log-rank tests for pairwise comparison. A two-sided P-value < 0.05 was considered statistically significant. Statistical analyses were performed using R version 4.3.1 (R Foundation for Statistical Computing, Vienna, Austria).

## Results

### Study population

A total of 319 patients met the inclusion criteria and were categorized into three groups: AF (*n* = 71), NSR (*n* = 213), and JR (*n* = 35) (Supplementary Fig. 1). The median follow-up duration was 56 months (range, 4–178 months).

The baseline characteristics of the three groups are summarized in Table 1. The mean age was 64.11 ± 7.89, 62.35 ± 9.75, and 66.8 ± 6.76 years in the AF, NSR, and JR groups, respectively (AF vs. NSR, *P* = 0.13; NSR vs. JR, *P* = 0.001; AF vs. JR, *P* = 0.07). There were no significant differences in sex distribution, diabetes mellitus, coronary artery disease, or AF type among the three groups. Persistent AF was the predominant type across all groups without significant differences (AF: 84.5%, NSR: 76.1%, JR: 82.9%).

After IPTW adjustment, the effective sample sizes were 58.7 for the AF group, 192.3 for the NSR group, and 23.5 for the JR group (Table 2). Standardized mean differences for all covariates were substantially reduced after weighting. The weighted distributions of AF type, preoperative LVEF, LAVI, and LVEDD demonstrated improved balance across all pairwise comparisons (Supplementary Fig. 2).

**Table 1:** Patients’ characteristics

Variable	AF (*N* = 71)	NSR (*N* = 213)	JR (*N* = 35)	*P*-value		
				AF vs. NSR	NSR vs. JR	AF vs. JR
Age, years	64.11 ± 7.89	62.35 ± 9.75	66.8 ± 6.76	0.13	***0.001***	0.07
Body surface area, m²	1.76 ± 0.23	1.67 ± 0.19	1.64 ± 0.18	***0.003***	0.32	***0.003***
Hospital stays, days	14.42 ± 5.15	13.62 ± 10.09	18.4 ± 10.48	0.38	***0.02***	***0.04***
Sex, male (%)	32 (45.1%)	103 (48.4%)	18 (51.4%)	0.63	0.74	0.54
**Concomitant procedure**						
Mitral valve repair	23 (32.4%)	64 (30%)	9 (25.7%)	0.71	0.60	0.48
Mitral valve replacement	16 (22.5%)	77 (36.2%)	10 (28.6%)	***0.03***	0.38	0.50
Tricuspid annuloplasty	48 (67.6%)	95 (44.6%)	18 (51.4%)	***< 0.001***	0.45	0.11
Aortic valve replacement	8 (11.3%)	42 (19.7%)	1 (2.9%)	0.11	***0.02***	0.28
CABG	5 (7%)	17 (8%)	4 (11.4%)	0.80	0.73	0.70
Persistent AF, n (%)	60 (84.5%)	162 (76.1%)	29 (82.9%)	0.14	0.38	0.83
Fine f-wave	54 (76.1%)	100 (46.9%)	26 (74.3%)	***< 0.001***	***0.003***	0.84
Preoperative AF	67 (94.4%)	185 (86.9%)	28 (80%)	0.08	0.41	0.05
**Comorbidities**						
Heart failure	14 (19.7%)	72 (33.8%)	8 (22.9%)	***0.025***	0.20	0.71
Coronary artery disease	9 (12.7%)	18 (8.5%)	5 (14.3%)	0.29	0.43	0.94
Diabetes mellitus	12 (16.9%)	41 (19.2%)	6 (17.1%)	0.66	0.77	0.98
Hypertension	41 (57.7%)	92 (43.2%)	13 (37.1%)	***0.033***	0.50	***0.05***
COPD	1 (1.4%)	8 (3.8%)	1 (2.9%)	0.56	0.82	0.81
Chronic renal failure	0 (0%)	8 (3.8%)	4 (11.4%)	0.21	0.13	***0.02***
History of stroke	11 (15.5%)	28 (13.1%)	9 (25.7%)	0.62	0.05	0.21
CHA₂DS₂-VASc score	2.42 ± 1.71	2.41 ± 1.68	2.94 ± 1.67	0.98	0.12	0.18
Redo sternotomy	2 (2.8%)	7 (3.3%)	3 (8.6%)	0.85	0.31	0.41
LAA preservation	30 (42.3%)	105 (49.3%)	19 (54.3%)	0.30	0.58	0.24
ACC time (min)	116.59 ± 29.36	116.35 ± 34.15	109.97 ± 41.31	0.95	0.41	0.41
CPB time (min)	167.69 ± 42.34	159.48 ± 44.38	159.18 ± 49.58	0.17	0.97	0.40

Significant p-values are shown in bold italics. CABG, coronary artery bypass graft; COPD, chronic obstructive pulmonary disease; AF, atrial fibrillation; NSR, normal sinus rhythm; JR, junctional rhythm; LAA, left atrial appendage; ACC, aortic cross-clamp; CPB, cardiopulmonary bypass

IPTW covariates: AF type, preoperative LVEF, preoperative LAVI, preoperative LVEDD

**Table 2:** Results of the inverse probability treatment weighting

Group	Before IPTW (*N*)	After IPTW (stabilized, weighted *N*)
Atrial fibrillation	69	58.7
Normal sinus rhythm	206	192.3
Junctional rhythm	34	23.5
**Total**	**309**	**274.5**

IPTW, inverse probability treatment weighting. After IPTW values represent effective sample sizes (ESS)

IPTW covariates: AF type, preoperative LVEF, preoperative LAVI, preoperative LVEDD

### Cardiac reverse remodeling

**Fig. 1:**
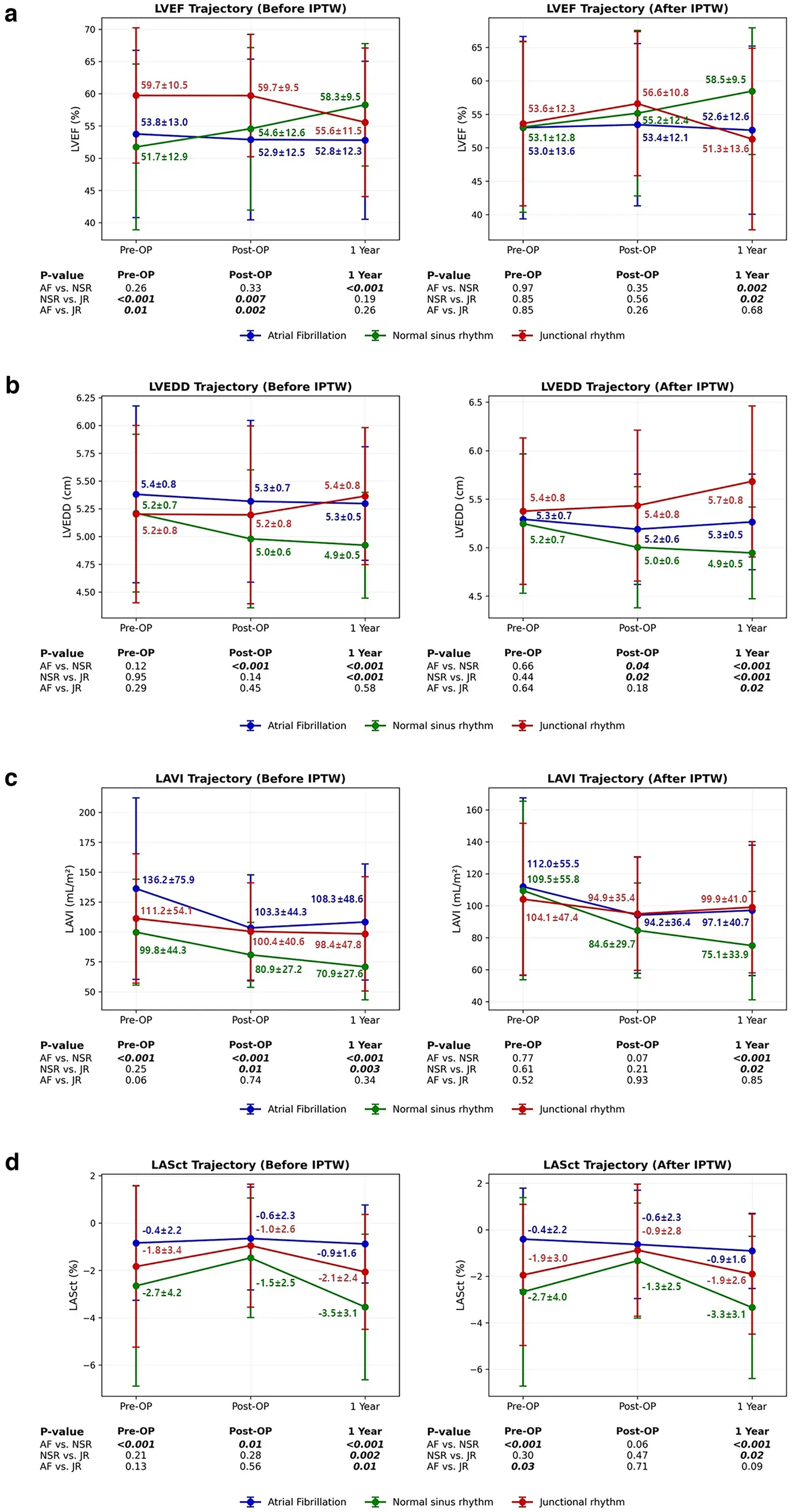
Before and after inverse probability of treatment weighting (IPTW)-adjusted echocardiographic trajectories across three time points (preoperative, early postoperative, and 1-year postoperative) for left ventricular ejection fraction (LVEF), left ventricular end-diastolic dimension (LVEDD), left atrial volume index (LAVI), and left atrial contractile strain (LASct). The normal sinus rhythm (NSR) group demonstrates progressive reverse remodeling from early postoperative to 1-year, while the junctional rhythm (JR) and atrial fibrillation (AF) groups show hemodynamic stagnation with minimal improvement over time

Echocardiographic trajectories were assessed at three time points—preoperative, early postoperative, and 1-year postoperative—to characterize the temporal pattern of reverse remodeling (Fig. 1). At the early postoperative time point, all three groups showed similar values across the major parameters, suggesting comparable immediate surgical effects. However, by 1-year follow-up, a clear divergence emerged: the NSR group demonstrated progressive reverse remodeling, whereas the JR and AF groups showed hemodynamic stagnation.

After IPTW adjustment, preoperative LVEF was comparable across the three groups (AF 53.0 ± 13.6%, NSR 53.1 ± 12.8%, JR 53.6 ± 12.3%; AF vs. NSR, *P* = 0.97; NSR vs. JR, *P* = 0.85; AF vs. JR, *P* = 0.85) (Fig. 1A). At one year, the NSR group showed significant improvement in LVEF (58.5 ± 9.5%), which was significantly higher than both the AF group (52.6 ± 12.6%; *P* = 0.002) and the JR group (51.3 ± 13.6%; *P* = 0.02), whereas the AF and JR groups did not differ (*P* = 0.68). This pattern was mirrored in the change over one year (ΔLVEF), which was significantly greater in the NSR group (5.37 ± 12.95%) than in the AF group (− 0.39 ± 10.94%; *P* = 0.001) or the JR group (− 2.31 ± 10.18%; *P* = 0.002), with no significant difference between the AF and JR groups (*P* = 0.45) (Table 3).

Left ventricular and atrial dimensions followed the same trajectory. Preoperative LVEDD was comparable across groups (AF 5.3 ± 0.7, NSR 5.2 ± 0.7, JR 5.4 ± 0.8 cm) (Fig. 1B), but at one year only the NSR group achieved significant reduction (4.9 ± 0.5 cm), a value lower than the AF group (5.3 ± 0.5 cm; *P* < 0.001) and markedly lower than the JR group (5.7 ± 0.8 cm; *P* < 0.001); a significant difference was also observed between the AF and JR groups (*P* = 0.02). Accordingly, the relative change in LVEDD over one year reflected a 4.54 ± 12.28% reduction in the NSR group, in contrast to a 0.59 ± 12.25% increase in the AF group and a 6.65 ± 13.17% increase in the JR group (AF vs. NSR, *P* = 0.006; NSR vs. JR, *P* < 0.001; AF vs. JR, *P* = 0.06).

LAVI showed a similar divergence: preoperative values were comparable (AF 112.0 ± 55.5, NSR 109.5 ± 55.8, JR 104.1 ± 47.4 mL/m²) (Fig. 1C), yet at one year the NSR group alone underwent significant reverse remodeling to 75.1 ± 33.9 mL/m², significantly lower than the AF group (97.1 ± 40.7 mL/m²; *P* < 0.001) and the JR group (99.1 ± 41.0 mL/m²; *P* = 0.02), with no difference between AF and JR (*P* = 0.85). The relative change in LAVI over one year confirmed this, with a 25.29 ± 24.19% reduction in the NSR group compared with a modest 8.69 ± 24.15% decrease in the AF group and a 1.38 ± 23.73% increase in the JR group (AF vs. NSR, *P* < 0.001; NSR vs. JR, *P* < 0.001; AF vs. JR, *P* = 0.11) (Table 3).

The same dissociation was most evident for left atrial contractile strain (LASct), the primary functional endpoint representing the “booster pump” function of the left atrium (Fig. 1D). At one year, the NSR group demonstrated the most pronounced recovery of LASct (− 3.3 ± 3.1%), which was significantly more negative—indicating stronger contraction—than both the AF group (− 0.9 ± 1.6%; *P* < 0.001) and the JR group (− 1.9 ± 2.6%; *P* = 0.02). Importantly, no significant difference in LASct was observed between the AF and JR groups (*P* = 0.09), indicating that JR failed to recover mechanical atrial function and mirrored the hemodynamic stagnation seen in the AF group (Table 3).

**Table 3:** Results of cardiac remodeling after the Cox–Maze procedure (before IPTW: Welch’s t-test, after IPTW: weighted Welch’s t-test)

Variables	AF	NSR	JR	*P*-value		
				AF vs. NSR	NSR vs. JR	AF vs. JR
**Before IPTW**						
ΔLVEF	-0.8 ± 11	6.5 ± 12.6	-4.1 ± 10.5	**< 0.001**	**< 0.001**	0.13
Relative ΔLVEDD	0 ± 12.6	-4.4 ± 12	4.5 ± 11.9	**0.01**	**< 0.001**	0.08
Relative ΔLAVI	-12.9 ± 24.4	-23.2 ± 23.9	-5.9 ± 24.7	**0.003**	**< 0.001**	0.19
1-year LASct	-0.9 ± 1.6	-3.5 ± 3.1	-2.1 ± 2.4	**< 0.001**	**0.002**	**0.01**
**After IPTW**						
ΔLVEF	-0.39 ± 10.94	5.37 ± 12.95	-2.31 ± 10.18	**0.001**	**0.002**	0.45
Relative ΔLVEDD	0.59 ± 12.25	-4.54 ± 12.28	6.65 ± 13.17	**0.006**	**< 0.001**	0.06
Relative ΔLAVI	-8.69 ± 24.15	-25.29 ± 24.19	1.38 ± 23.73	**< 0.001**	**< 0.001**	0.11
1-year LASct	-0.91 ± 1.62	-3.34 ± 3.06	-1.91 ± 2.58	**< 0.001**	**0.02**	0.09

Significant p-values are shown in bold italics. IPTW, inverse probability treatment weighting; AF, atrial fibrillation; NSR, normal sinus rhythm; JR, junctional rhythm; LVEF, left ventricular ejection fraction; LVEDD, left ventricular end-diastolic dimension; LAVI, left atrial volume index; LASct, left atrial contractile strain. ΔLVEF = (1-year LVEF – preoperative LVEF). Relative ΔLVEDD = [(1-year LVEDD − preoperative LVEDD) / preoperative LVEDD] × 100. Relative ΔLAVI = [(1-year LAVI − preoperative LAVI) / preoperative LAVI] × 100. LVEF and LASct were measured in percentages (%), LVEDD in centimeters (cm), and LAVI in mL/m². All continuous variables are expressed as mean ± standard deviation

### Clinical outcomes

**Fig. 2:**
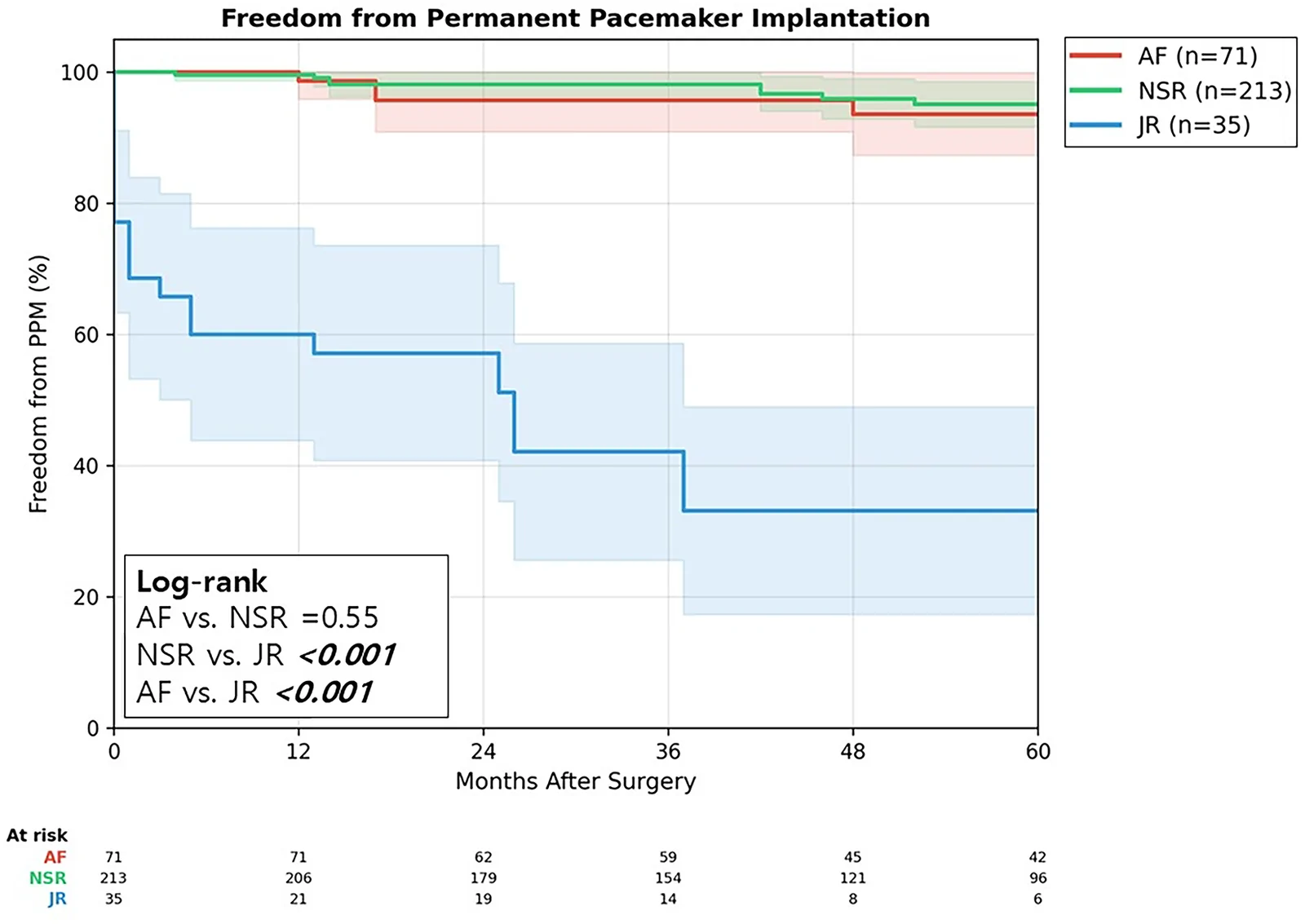
Kaplan–Meier analysis of freedom from permanent pacemaker (PPM) implantation with 95% confidence intervals and number of patients at risk. Pairwise log-rank P-values are shown for all three comparisons (AF vs. NSR, NSR vs. JR, AF vs. JR)

The most striking clinical difference among the three groups was the need for permanent pacemaker (PPM) implantation (Fig. 2). The JR group had a dramatically higher cumulative rate of PPM implantation at 1, 3, and 5 years (40.0%, 57.9%, and 66.9%, respectively) than either the AF group (1.4%, 4.4%, and 6.5%) or the NSR group (0.5%, 1.9%, and 5.0%). Within the JR group, 8 patients (22.9%) required early PPM implantation (within 30 days) and 18 patients (51.4%) required late implantation. Kaplan–Meier analysis confirmed significantly lower freedom from PPM in the JR group, whereas the AF and NSR groups did not differ (log-rank: AF vs. NSR, *P* = 0.549; NSR vs. JR, *P* < 0.001; AF vs. JR, *P* < 0.001).

**Fig. 3:**
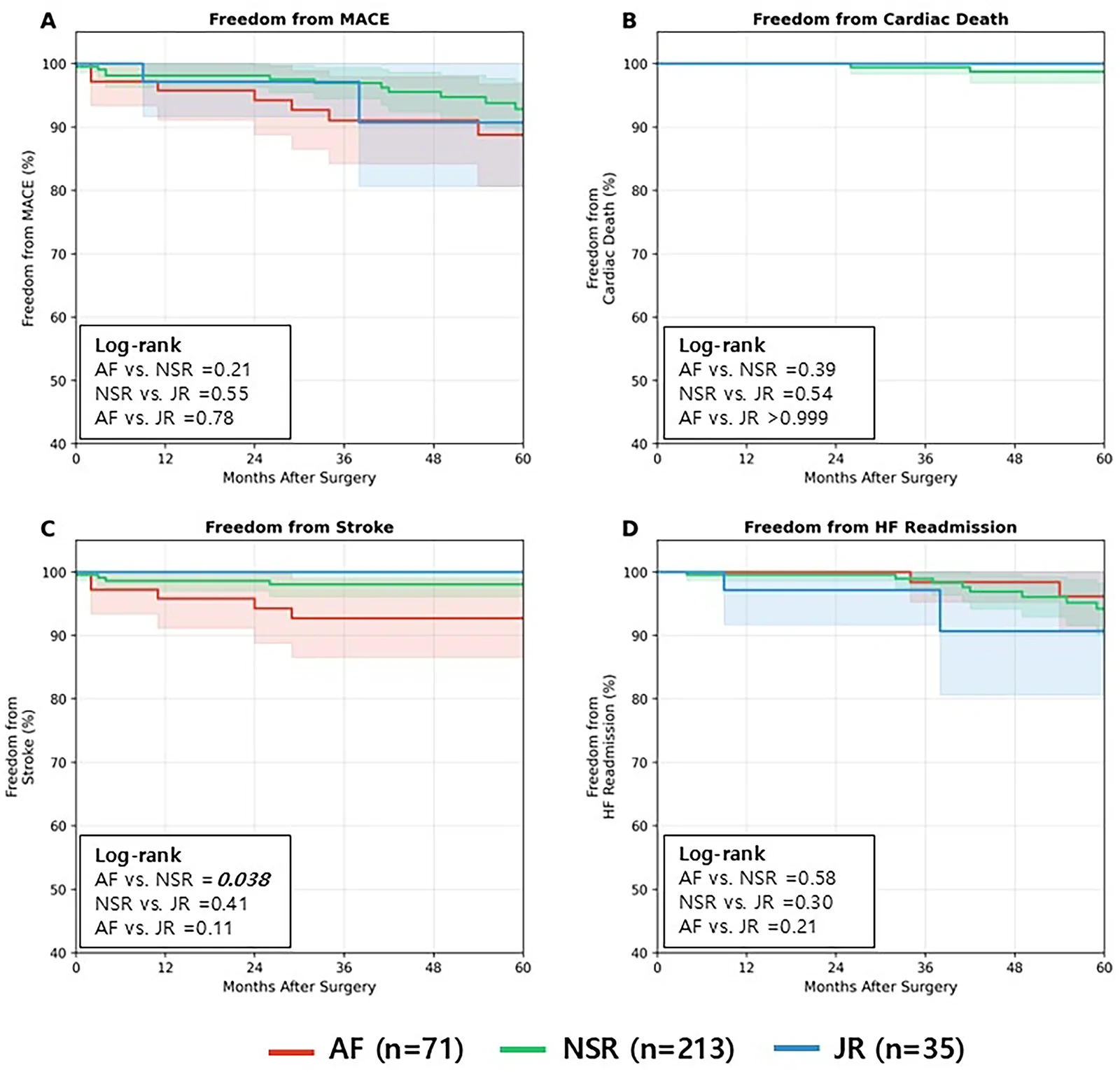
Kaplan–Meier analysis of freedom from major adverse cardiac events (MACE), defined as the composite of stroke, heart failure requiring hospitalization, and cardiac death, with 95% confidence intervals and number of patients at risk. Pairwise log-rank P-values are shown for all three comparisons

In contrast to this divergence in pacemaker dependency, major adverse cardiac events (MACE)—defined as the composite of stroke, heart failure requiring hospitalization, and cardiac death—did not differ significantly among the groups (Fig. 3). MACE occurred in 10 patients (14.1%) in the AF group, 20 (9.4%) in the NSR group, and 3 (8.6%) in the JR group (*P* = 0.497), and Kaplan–Meier analysis showed no significant difference (log-rank: AF vs. NSR, *P* = 0.207; NSR vs. JR, *P* = 0.547; AF vs. JR, *P* = 0.776). The individual components were likewise comparable across groups. Stroke occurred in 5 AF (7%), 7 NSR (3.3%), and 0 JR patients (0%) (log-rank: AF vs. NSR, *P* = 0.038; NSR vs. JR, *P* = 0.409; AF vs. JR, *P* = 0.111); heart failure readmission occurred in 4 AF (5.6%), 15 NSR (7%), and 3 JR patients (8.6%) (log-rank: AF vs. NSR, *P* = 0.580; NSR vs. JR, *P* = 0.301; AF vs. JR, *P* = 0.207); and cardiac death occurred in 2 AF (2.8%), 2 NSR (0.9%), and 0 JR patients (0%) (log-rank: AF vs. NSR, *P* = 0.387; NSR vs. JR, *P* = 0.535; AF vs. JR, *P* = 1.000). None of the individual components reached statistical significance among the three groups.

## Discussion

This study provides compelling evidence that junctional rhythm after the Maze procedure is not a benign alternative to normal sinus rhythm. By employing a strict rhythm-stable cohort design and utilizing IPTW-adjusted analyses, we demonstrate three key findings: (1) JR fails to achieve cardiac reverse remodeling, behaving hemodynamically similar to persistent AF; (2) JR carries a dramatically elevated risk of permanent pacemaker implantation; and (3) LASct recovery is exclusively observed in the NSR group, underscoring the critical importance of true sinus rhythm restoration.

The most striking finding of this study is the hemodynamic equivalence between the JR and AF groups. While the NSR group demonstrated significant improvements across all remodeling parameters—LVEF, LAVI, LVEDD, and LASct—the JR group showed no such recovery. This observation challenges the conventional binary classification of post-Maze outcomes, where JR is typically grouped with NSR as a “successful” outcome [3, 4]. The hemodynamic stagnation in the JR group can be attributed to the loss of coordinated atrial contraction, a hallmark of junctional rhythm that directly mirrors the pathophysiology of AF [17]. Without effective atrial systole, the “atrial kick” is absent, resulting in reduced ventricular filling and impaired cardiac output.

Our use of LASct as a primary endpoint represents a methodological advance in evaluating post-Maze outcomes. Traditional metrics such as LVEF and LAVI, while useful, are influenced by loading conditions and may not fully capture the nuanced recovery of atrial mechanical function [18]. LASct, measured by speckle-tracking echocardiography, directly quantifies the “booster pump” function of the left atrium—the active contractile phase that is inherently dependent on organized atrial depolarization. In our IPTW-adjusted analysis, only the NSR group showed significant recovery of LASct, while both the JR and AF groups exhibited minimal contractile function. This finding underscores that electrical rhythm alone (absence of AF) is insufficient; true mechanical recovery requires coordinated sinus node-driven atrial contraction [19].

The dramatically elevated PPM implantation rate in the JR group (66.9% at 5 years, 74.3% over the entire follow-up period) compared to the NSR (5.0% at 5 years) and AF groups (6.5% at 5 years) is clinically significant and warrants particular attention. This finding is consistent with the known pathophysiology of JR, which reflects underlying sinus node dysfunction or conduction system disease that may be exacerbated by surgical ablation [20, 21]. The high rate of both early (22.9%) and late PPM implantation (51.4%) in the JR group suggests that junctional rhythm is not merely a transient postoperative phenomenon but often represents a permanent conduction abnormality requiring device therapy [22].

Interestingly, despite the adverse hemodynamic profile of JR, the rate of MACE did not differ significantly among the three groups. This may be explained by the relatively short follow-up period and the protective effect of appropriate medical management and timely PPM implantation in the JR group. However, the absence of cardiac reverse remodeling in the JR group raises concern for long-term adverse outcomes that may manifest beyond the observation period of this study.

These findings carry important clinical implications. First, JR should be explicitly differentiated from NSR in reporting post-Maze outcomes, rather than being aggregated into a single “sinus conversion” category [23]. Second, patients who develop JR after the Maze procedure should be monitored closely for hemodynamic compromise and the need for PPM implantation. Third, pre-operative predictors of JR—which may include advanced age, pre-existing conduction disease, and extensive atrial remodeling—should be identified to enable risk stratification and informed surgical decision-making.

### Limitations

This study has several limitations. First, the retrospective, single-center design inherently limits the generalizability of our findings. Second, the strict inclusion of rhythm-stable patients may introduce selection bias by excluding patients with transient or evolving rhythm disturbances. Third, while IPTW adjusts for measured confounders, residual confounding from unmeasured variables cannot be excluded. Fourth, the JR group was relatively small (*n* = 35), which may limit statistical power for detecting differences in MACE outcomes. Fifth, because electrophysiological and atrial pacing-capture testing was not performed, sick sinus syndrome and atrial standstill could not be fully distinguished in the JR group. Finally, in patients who received a permanent pacemaker during the study period, the transthoracic echocardiographic measurements were obtained after pacemaker implantation; consequently, the influence of pacing on cardiac function could not be entirely excluded.

## Conclusions

Junctional rhythm after the Maze procedure is not a benign alternative to normal sinus rhythm. It mimics the hemodynamic profile of atrial fibrillation, failing to achieve cardiac reverse remodeling and carrying a dramatically elevated risk of permanent pacemaker dependency. Left atrial contractile strain recovery is exclusively observed in the NSR group, highlighting the critical importance of true sinus rhythm restoration for meaningful functional recovery. JR should be clinically differentiated from NSR and recognized as a suboptimal hemodynamic outcome. These findings advocate for a paradigm shift in defining surgical success after the Maze procedure, moving beyond simple “freedom from AF” to incorporate mechanical atrial recovery as a key outcome measure.

## Supplementary Information

Below is the link to the electronic supplementary material.

Supplementary Material 1: Patient selection flow diagram.

Supplementary Material 2: Covariate balance before and after inverse probability of treatment weighting (IPTW) adjustment (Love Plot). Standardized mean differences (SMD) for all covariates are displayed, with the dashed line indicating the threshold of adequate balance.

## Data Availability

No datasets were generated or analysed during the current study.

## Abbreviations

AF: Atrial fibrillation
ASE: American Society of Echocardiography
CABG: Coronary artery bypass graft
COPD: Chronic obstructive pulmonary disease
CPB: Cardiopulmonary bypass
ACC: Aortic cross-clamp
ECG: Electrocardiogram
ESC: European Society of Cardiology
IPTW: Inverse probability of treatment weighting
JR: Junctional rhythm
LA: Left atrial
LAA: Left atrial appendage
LAD: Left atrial diameter
LAS: Left atrial strain
LAScd: Left atrial conduit strain
LASct: Left atrial contractile strain
LASr: Left atrial reservoir strain
LAVI: Left atrial volume index
LVEDD: Left ventricular end-diastolic dimension
LVEF: Left ventricular ejection fraction
MACE: Major adverse cardiac events
NSR: Normal sinus rhythm
PPM: Permanent pacemaker
RA: Right atrial
SMD: Standardized mean differences
STE: Speckle-tracking echocardiography
TTE: Transthoracic echocardiography
